# Why Does Rubin's Vase Differ Radically From Optical Illusions? Framing Effects *Contra* Cognitive Illusions

**DOI:** 10.3389/fpsyg.2021.597758

**Published:** 2021-09-21

**Authors:** Elias L. Khalil

**Affiliations:** School of Public Administration and Development Economics, Doha Institute for Graduate Studies, Doha, Qatar

**Keywords:** behavioral economics, behavioral decision sciences, prospect theory, reference points, reference-as-heuristic, reference-as-context, perspective, Kuhn's paradigm

## Abstract

Many researchers use the term “context” loosely to denote diverse kinds of reference points. The issue is not about terminology but rather about the common conflation of one kind of reference points, such as rules of perception, which is responsible for optical illusions, with another kind, known as “context” or “frame,” as exemplified in Rubin's vase. Many researchers regard Rubin's vase as a special kind of optical illusions. This paper rather argues that the two phenomena are radically different. Optical illusions are occasional mistakes that people quickly recognize and eagerly correct, while the different figures of Rubin's vase are not mistakes but, rather, the outcomes of different perspectives that do not need correction. The competing figures in Rubin's vase can, at best, in light of more information, be more warranted or unwarranted. This paper discusses at length one ramification of the proposed distinction. The framing effects, such as loss/gain frame, are the products of contexts and, hence, resemble greatly the figures in Rubin's vase. In contrast, cognitive illusions generated occasionally by the rules of thumb (heuristics) are mistakes and, hence, resemble optical illusions. The proposed distinction carries other ramifications regarding, e.g., happiness studies, moral judgments, and the new philosophy of science.

## Introduction

Consider these two valuations that you may undertake:

1. You train diligently for many years and expect to win the “first rank” in a marathon race, but instead, you win the second out of three possible ranks.
2. You value a radio priced at $498 as being much cheaper than an identical radio priced at $501.

In valuation (1), you use the “first rank” as a *context* acting as the reference point to evaluate the actual outcome, while in valuation (2), you use the “$400s” category as what this study calls “*relativeness*” acting as the reference point to evaluate the price of the radio. The context, or what is also called the “frame” in this paper, and relativeness, or what is also called the “heuristic,” can be considered reference points in the generic sense. However, do they have an equivalent function? Is the evaluation of accomplishment analytically equivalent to the evaluation of a price *via* relative comparisons (see Gigerenzer and Goldstein, 1996)? Stating the research question differently,

**The research question:** Is the reference point acting as a context (frame) to evaluate the achievement more or less indistinguishable from the reference point acting as relativeness (heuristic) needed to assess the price of a good?

This paper proposes the following answer:

**Core hypothesis:** the reference point providing a context to make sense of the achievement belongs to a different genus than the reference point providing relativeness acting as a benchmark or an anchor used to assess the price of a good.

While the choice of the terms “context” (frame) and “relativeness” (heuristic) might be debatable, the issue is not about the lexicon. The issue is rather about whether they denote radically different genera of reference points, as the research question registers. This study demonstrates that there are rather two radically different genera of reference points, as the core hypothesis registers.

As for the reference point suggested by the “$498” price, the price of “$498” instead of “$501” amounts to a priming effect, i.e., the subtle suggestion that the price falls within the “$400s” category rather than the “$500s” category. The decision maker (DM) would normally think that $498 is much cheaper than $501 as much as the average of the former category, $450, is cheaper than the average of the latter, $550. Putting it differently, the “$400” category acts as shorthand or fast indication of the price, i.e., it acts as heuristics. The heuristics in this function reveals “relativeness” in the sense of facilitating the cognitive comparison of the item of purchase product relative to alternatives whose cost falls within the $400s range. The product, hence, would definitely appear more attractive than if it was priced at $501, where the consumer would perceive it as about $550.

As for the reference point suggested by the “first rank” belief, it acts as a frame or context revealing the meaning of the outcome. This meaning allows the DM to make sense of his or her actual achievement.

This study advances primary and secondary theses. The primary thesis extends the core hypothesis articulated above. Namely, the reference point acting as a context occasions framing effects such as the loss/gain frame effects. Such framing effects are not the subject of correction, while, as detailed below, the framing effects are the outcome of a context (frame) that functions as a viewpoint or a perspective. As such, the context cannot be empirically based, i.e., it cannot be confirmed by appealing to the facts. The frame is rather how the mind of the DM organizes the facts into some kind of coherence, which provides meaning to them.

In contrast, the reference point acting as relativeness occasions rules of thumb such as stereotypes, generalizations, or in short heuristics. Such rules of thumb may, on some occasions, fail. When they do, the outcomes are errors of judgment, what are called generally “cognitive illusions.” While framing effects cannot be the subject of correction, cognitive illusions are expressly the subject of correction. So, the primary thesis derived from the core hypothesis is that framing effects differ radically from cognitive illusions that arise occasionally from the use of relativeness, namely, the rules of thumb or heuristics that are representative of the class of phenomena under focus. This paper explains why the two sets of phenomena belong to different genera.

**Figure 1:**
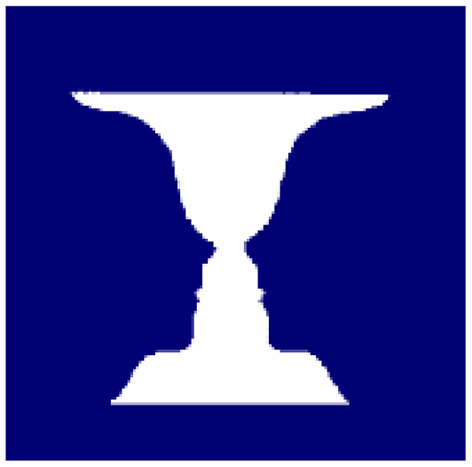
Plain Rubin's vase (https://psychology.wikia.org/wiki/Rubin_vase—retrieved 6/6/2020).

A similar difference seems to set Rubin's vase apart from optical illusions. As detailed below (see Figure 1), Rubin's vase is an example of how figures or images change depending on a viewpoint, perspective, background, or in short, a context. If the context is the blue pixels, the DM sees a vase. If the context is the white pixels, the DM sees two opposed faces in a profile. There is no correct or incorrect figure.

The situation differs from optical illusions. As detailed below, what the DM sees is clearly incorrect, and he or she embarrassingly tries to correct the judgment. When the DM sees, as a result of an interference, for instance, that one line is longer than another, when in fact, they are of equal length, what is operative is not the viewpoint or context. Rather, it is an interference in the visual field that causes mistaken neural processes.

For instance, Porciello et al. (2018) show that, in almost all occasions, DMs recognize their own face in the mirror. However, DMs may make mistakes on some occasions. They may process the stimuli regarding the face of another person and judge it as their own, what is known as the “enfacement illusion” (see also Tajadura-Jiménez et al., 2012; Bufalari et al., 2015). Ishizu (2013) explains that the brain often works with ambiguous bits of information about a human face. The brain disambiguates the foggy bits into a full face, which is usually that of the person, especially if prompted by the experimenter (priming) in this direction.

Decision scientists recognize the difference between such illusions and Rubin's vase. However, as this paper registers, it is insufficient to recognize the difference. We need to answer the question: What is the basis of the difference? Is Rubin's vase, basically, one variant of optical illusions, alongside many other variants—or are both radically different? This study argues that Rubin's vase does differ *radically* from optical illusions. While Rubin's vase, ultimately, cannot be the subject of correction, optical illusions are the occasional failures of rules of perception—failures that are the subject of correction.

This leads to the consequent secondary thesis. Namely, the fissure that sets Rubin's vase apart from optical illusions in the case of vision, is the same fissure that sets framing effects apart from cognitive illusions in the case of decision-making.

Table 1 acts as the compass of this paper, summing up two questions. The first is whether there is a difference between the two operants, context contra relativeness. According to the primary thesis of this study, the context operant is radically different than the relativeness operant. The second question is whether there is a difference between the vision and decision sciences regarding the operation of context as opposed to relativeness. According to the secondary thesis, there is no difference. The context/relativeness distinction is isomorphic, i.e., cuts across both sciences. Put differently, the real difference is between the two operants. Rubin's vase and framing effects are part of the same phenomenon informed by the context operant, while optical illusions and cognitive illusions are part of another phenomenon informed by the relativeness operant.

**Table 1:** Where does the difference really lie?.

**Do the Operants Differ?Do the Sciences Differ?**	**Context is the operant**	**Relativeness is the operant**
Vision Science	Rubin's Vase	Rules of Perception —–> Optical Illusions *when such rules fail*
Decision Science	Framing Effects	Rules of Thumb (Heuristics)–> Cognitive Illusions *when such rules fail*

It is not common for empirical work in the behavioral science literature to take up the task of directly distinguishing between context (framing effects) and relativeness (heuristics). Nonetheless, there is tacit recognition in the literature that the two phenomena are different. However, this difference is not sharply articulated—a challenge that this study takes. Additionally, the difference is usually obscured when mentioned alongside many unrelated behavior biases such as inequity aversion as exhibited in the ultimatum game, the avoidance of temptations *via* commitments and ethical rules, and what one may call “anxiety aversion” that surrounds ambiguity that might lie behind the certainty effect or what is called the “Allais Paradox” (see Camerer, 2003; Gintis, 2009; Dhami, 2017; Khalil, unpublished). This study does not discuss these behavioral biases, given the primary thesis regarding the context/relativeness fissure.

The goal of this study is to establish, in a systematic manner, the context/relativeness fissure. The first section encapsulates the argument of the paper. The succeeding two sections focus on vision science, demonstrating the context/relativeness fissure thesis by contrasting Rubin's vase and optical illusions, respectively. The following two sections focus on decision sciences, demonstrating the same thesis by contrasting framing effects and cognitive illusions, respectively. The last section highlights a few payoffs of the proposed context/relativeness distinction, e.g., regarding happiness studies, moral judgments, and the new the philosophy of science.

## The Paper in a Nutshell

### Rubin's Vase (Context) vs. Optical Illusions (Relativeness)

Examples of the Rubin's vase phenomenon include Rubin's vase itself, Schröder staircase, and the Necker cube (Kornmeier and Bach, 2005)^1^. The Necker cube differs from the other two. However, they share a defining feature: this genus of reference points is about the alteration of perspective. As detailed below, while the perspective alteration in Rubin's vase and Schröder staircase is the outcome of the “figure-ground” contrast, the perspective alteration in the Necker cube is the outcome of an orientation switch.

In general, a perspective can be defined as the way to process information about the *whole* field (see Hasson et al., 2001), allowing the DM to adopt different ways. The holistic information processing is the essential contribution of Edgar Rubin after whom this genus of reference points is named (see Pind, 2015).

Examples of optical illusions include the Müller-Lyer illusion (**Figure 4**), the Ponzo illusion (see Shapiro and Todorović, 2017), and the lightness illusion (**Figure 5**). The defining feature of this genus of reference points is the processing of *local* information pertaining to each segment of the visual field, whereas cognitive activity assembles the segmented parts into a perception (Hasson et al., 2001). Such a perception fails in some peculiar circumstances—where DMs sense such failure as an optical illusion.

There is hardly any vision researcher who outright conflates Rubin's vase and optical illusions. Vision researchers generally recognize that there is a difference. Some of them even explicitly emphasize it. For instance, Todorović (2020) finds that the main difference lies in the fact that optical illusions involve errors, while Rubin's vase does not:

In other words, the notion of context effects may be more general than the notion of illusions. In the following, illustrations of three phenomena [resembling Rubin's vase] are presented, which involve strong context effects but do not quite fit the illusion scheme because they do not seem to involve errors (Todorović, 2020, p. 1183).

While the “error” criterion is important, we should not stop with this difference. Otherwise, researchers would slip back and regard Rubin's vase as a particular variety of optical illusions. Indeed, in the same paper, Todorović uses a lexicon—when he calls optical illusions the result of “context” while Rubin's vase the result of “strong context” —suggesting that the two phenomena lie along a continuum.

If we view the two phenomena as different only in scale, it is a short step to explaining perspective alteration as a “temporary” optical illusion, as it would disappear once information ambiguity vanishes (Kornmeier and Bach, 2005; Macpherson, 2006). The idea that Rubin's vase is the outcome of information poverty is deep-seated in the literature. As shown below, the literature starts with an unexamined entry-point regarding perspectives to be like all other beliefs, namely, as derived ultimately from empirical facts. The literature, consequently, sees that, once the information poverty vanishes, such as in the case of touching and feeling the figure, say, a vase, the DM would embrace the judgment that the object is a vase, while considering the alternative figure, i.e., the two opposed faces in a profile, as “incorrect.”

This paper disputes such an entry point. A perspective is not a belief that can be traced back to information or empirical facts and, hence, can be changed *via*, e.g., Bayesian updating (Khalil, 2010). It is rather a context that the DM imagines to order the data in a collective way, i.e., to facilitate holistic information processing as Rubin has envisioned. Once we dispense with the entry point of the literature, the difference between Rubin's vase and rules of perception goes deeper than the “error” criterion. The difference is the recognition of the role of perspective or context with respect to one genus of cognition involving holistic information processing while absent in a genus involving only local information processing.

It is common for researchers to suppose that optical illusions arise from perspectives—in the same manner as Rubin's vase. This is probably the case because they appear to be similar. For example, the Ponzo illusion, which is the product of the distance-size invariance heuristics (see Kaufman et al., 2007), appears to be the product of perspective. The distance-size invariance heuristics is a reasonable rule, namely, distanced objects are not as small as they seem. However, the heuristic fails in the Ponzo illusion case. The failure is the result of the set of the two railroad tracks on paper misleading the DM to suppose that objects are within a three-dimensional space and, consequently, to judge distant objects along the track as larger than they actually are. However, the use of the three-dimensional space heuristics cannot be a perspective, as the three-dimension construction is neither a ground in the actual Rubin's vase—nor orientation in the case of the Necker cube. If the three-dimensional space were a perspective, what is the alternative? The two-dimensional space cannot be, as it does not arise even when the information is minimal. The three-dimensional space is simply the wrong application of the heuristics in this case. Otherwise, the three-dimensional space is efficient.

Likewise, the Jastrow illusion is the failure of otherwise efficient heuristics. The rule of perception, namely, that the DM should compare the lengths of adjacent arches, is a quick and efficient rule to judge relative sizes (see Pick and Pierce, 1993).

### Framing Effects (Context) vs. Cognitive Illusions (Relativeness)

Regarding the example stated at the outset, the valuation of “second rank” winning takes the “first rank” as the frame or what can be generally called “context.” The context allows the DM to make sense of the “second rank,” i.e., of the actual well-being. The consequent satisfaction, which can be negative or positive, is the framing effect pertinent to happiness.

There are different varieties of framing effects. They include bonding/attachment, hopefulness, the loss/gain frame effects, and aspiration. This research only studies the loss/gain framing and refers to it with the generic term “framing effects.” One stark example of a framing effect is the famous Asian disease experiment of Tversky and Kahneman (1981) (see also Kahneman and Tversky, 1979; Tversky and Kahneman, 1986).

In contrast, the valuation of a radio against a benchmark, the “$400s,” acts as a point of relativeness or, in short, heuristics. The heuristics allows the DM to quickly evaluate whether the radio is cheap enough. The consequent satisfaction, which can be negative or positive, is well-being—i.e., utility in the pecuniary sense. The DM would reap a greater consumer surplus, thinking that he paid a price within the “$400s” category rather than the “$500s” category. There is no context involved, and, hence, there is no satisfaction other than well-being.

In our example, though, there is a cognitive illusion, a failure of the heuristic “$400s.” The perceived great gain of consumer surplus is illusory, given that the saving is only $2. On most occasions, however, the heuristic “$400s” is a rather fast and effective tool to process information with minimum cognitive cost.

To clarify the concept of “relativeness” or “heuristics,” it is general enough to encompass different varieties of mechanisms. The mechanism can be the elimination-by-aspects heuristics focusing on absolute thresholds (e.g., Tversky, 1972a,b), the take-the-best heuristics focusing on the relative comparison (e.g., Gigerenzer and Goldstein, 1996; White et al., 2015), and so on. The mechanism is greatly determined by the particular case. Nonetheless, all mechanisms are heuristics *per se* in so far, they are rules of thumb geared to minimize cognitive cost.

The main goal of this study is to uncover the difference between heuristics and contexts. While behavioral scientists generally recognize that they differ, and discuss them under different names, they do not distinguish them sharply enough. For instance, Kahneman (2011, Chs. 1–25) exposes various kinds of heuristics, particularly focusing on their failure in experiments. Meanwhile, he subtly advances his own concept of “mental economy,” which is roughly equivalent to the concept of the standard economist of “bounded rationality” and what psychologists call “cognitive economy” (e.g., Conrad, 1972; Kusev and Van Schaik, 2013)^2^. The concept is the application of cost-benefit analysis to cognitive processes. This analysis is suitable for explaining the origin and function of heuristics. However, Kahneman (2011, Ch. 26) uses dual process theory, the basis of the mental economy, to explain as well framing effects, such as his early work with Tversky on the Asian disease (Tversky and Kahneman, 1981)^3^.

Kahneman (2011, Chs. 25–26) even tries to explain framing effects *via* the psychophysics of sensations that are appropriate to making sense of optical illusions. The conflation is not limited to Kahneman. Behavioral decision scientists (e.g., Pothos and Busemeyer, 2013; see Khalil, 2013a) and behavioral economists (e.g., Munro and Sugden, 2003; Köszegi and Rabin, 2006) propose models regarding reference points while lumping both framing effects and cognitive illusions.

## Rubin's Vase

### Perspective Alteration

This study takes Rubin's vase as the epitome of a genus of effects arising from perspective alteration. As illustrated by the *plain* Rubin's vase (Figure 1), the perspective or viewpoint of the DM alternates, where the alteration gives rise to two or more figures, without any change of the sense data. The perspective alteration is about the figure-ground contrast: Which is the ground—the pigmented or non-pigmented pixels? It cannot be both.

While the figure-ground is not evident in the Necker cube, the perspective alteration appears in a different manner. It involves spatial orientation: What is the orientation—upward to the left-hand or downward to the right-hand? Similar to the case of the actual Rubin's vase where the background cannot, simultaneously, be both the blue and white pixels, the orientation cannot simultaneously be both upward and downward. As in the case of Rubin's vase where one cannot simultaneously see the vase and the alternative, the two opposed faces in a profile, one cannot simultaneously see the two locations of the red ball in Necker cube. In both cases, the reason is the same. It is impossible for the DM in Rubin's vase to see the vase unless he or she has already fixed the blue pixels as the ground. Likewise, it is impossible for the DM in the Necker cube to see the red ball in one corner, but not the other, unless he or she has already fixed the orientation.

To caution, however, the framing effect does not arise by the use of *any* imagined perspective. The imagined perspective must be pertinent to the sensory data—whether it is the pigmented pixels or the spatial orientation of lines. In the case of Rubin's vase, the ground is co-determined with the figure. In the case of the Necker cube, the spatial orientation is co-determined with the lines.

Such holistic co-determination defining the perspective is absent in rules of perception and their by-products, optical illusions. In the Jastrow illusion, e.g., the rule of perception is rather imported to the situation, i.e., it is a presupposed generalization from other experiences. It does not arise from some holistic information processing related to the particular case at hand.

### When the Ground Becomes Fixed: the Loss/Gain Frame

The rest of the paper considers Rubin's vase rather than the Necker cube, as the exemplar of the perspective alteration genus. Let us start with a plain Rubin's vase, the classic apotheosis of the figure-ground contrast (Figure 1). If the DM chose the dark pigmented area as the ground, the vase would be the figure. If instead, the DM chose the non-pigmented area as the ground, the two opposed faces in a profile would be the figure.

**Figure 2:**
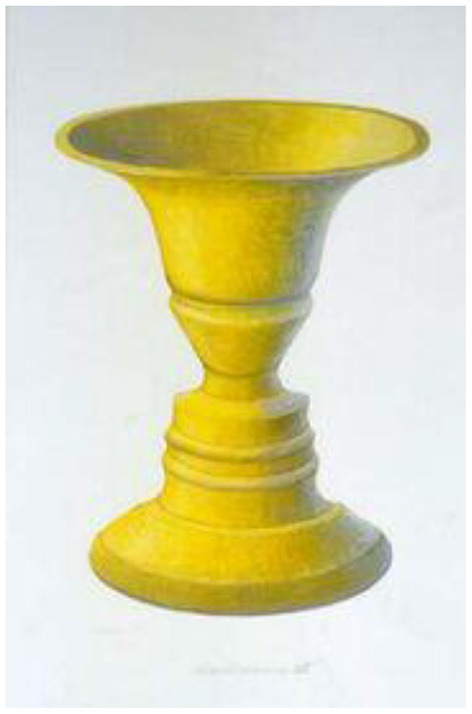
Gold-shaded Rubin's vase (https://psychology.wikia.org/wiki/Rubin_vase—retrieved 6/6/2020).

**Figure 3:**
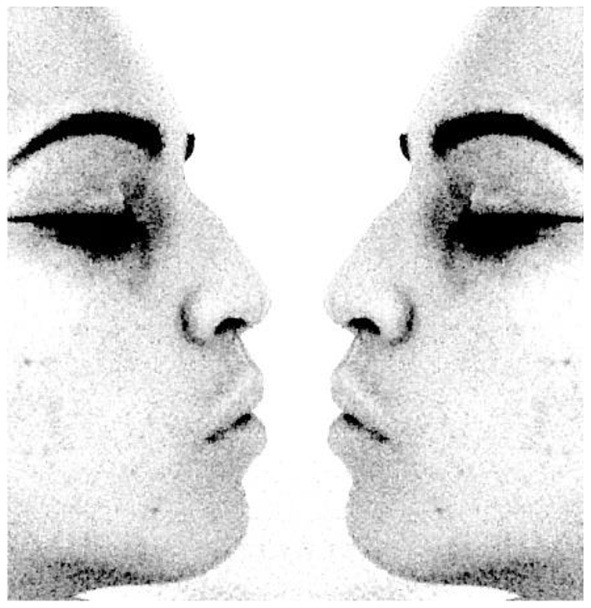
Human-expression Rubin's vase (http://ms-raz.com/rubins-vase–retrieved 13/6/2020).

In this plain representation of Rubin's vase, the DM would experience perspective alternation with equal probability. However, once Rubin's vase becomes enriched with more data, one perspective would become more warranted. As most DMs must agree, the gold-shaded Rubin's vase (Figure 2) gives more support to the perspective that the figure is a vase than the two opposed faces in a profile. Reversely, a Rubin's vase that is enriched with eyebrows and other facial details (Figure 3) gives more support to the perspective that the figure is two opposed faces in a profile than a vase. In both Figures 2, 3, the experimenter more or less fixes the ground, which prompts DMs to see one figure more frequently than the alternative.

This is similar to the Asian disease and other loss/gain frame experiments (Tversky and Kahneman, 1981). In these experiments, the experimenter more or less fixes the ground. In the between-subject design of the Asian disease experiment, if the experimenter fixes the loss frame as the ground, subjects tend to make risk-loving choices. If the experimenter fixes the gain frame as the ground, subjects tend to make risk-averse choices.

### What Is Remarkable About Rubin's Vase?

Once the experimenter fixes the ground with greater detail, e.g., adding eyebrows or, instead, golden shades in Rubin's vase—the DM would not experience perspective alteration, at least not in equal probability. What is remarkable about the Rubin's vase is that, despite the greater detail, the dominant perspective or gestalt cannot totally exclude the subdued alternative. The subdued alternative remains implicit, a phenomenon that can be called “entailment.” Figure 2 still entails, although much less clearly, the two opposed faces in a profile. Likewise, Figure 3 still entails, although with much greater difficulty, the vase.

The dwarfed two opposed faces in the profile entailed in Figure 2, or the doubtful vase entailed in Figure 3, cannot vanish even if they are unwarranted. If they vanish completely, there was no figure-ground contrast in the first place. Any warranted figure that is the outcome of a gestalt (perspective) must entail, evenly very dimly, the alternative figure.

One might suspect that the idea of entailment is artificial, the product of the two-dimension representation of Rubin's vase. Such a representation amounts to a controlled laboratory environment ruling out a three-dimensional experience, which allows the DM to see the object from all angles and, if pertinent, to touch, smell, hear, and taste it. In a non-controlled, three-dimensional experience, one might suspect there is no room for the entailment idea. That is, the Rubin's vase phenomenon is simply the product of impoverished two-dimensional experiences affording ambiguous information.

Such suspicion, as suggested above, stems from the entry-point supposition that all figures can be, as the case with ordinary objects, stripped from the ground in a non-laboratory setting. However, this is not necessarily the case. Even if the DM sees a vase on a table in the dining room, where he or she can go around it and touch it, the DM might experience a perspective alteration and see the alternative figure. It is true that such perspective alteration is very rare in the highly detailed Rubin's vase. Still, the alternative figure cannot be excluded in everyday encounters.

In this light, the laboratory-based two-dimensional Rubin's vase experience makes such rare events more common—particularly with plain Rubin's vase (Figure 1). That is, the laboratory setting is not “unreal” —but rather, an abstraction revealing a reality rarely detected as a result of confounding factors, i.e., the details that fix the ground.

This has one payoff regarding the philosophy of science (for a discussion of philosophy of science implications, see Appendix 2, from the author or available as “Supplementary Material” at the end of the paper). Even when the ground becomes more detailed, pivoting it as the dominant ground, it does not mean that the alternative ground (gestalt, perspective, or context) is incorrect. It only means that the alternative ground is “unwarranted.” Given that the alternative is entailed, the entailment idea leads to the distinction between “incorrectness” and “un-warrantness.” Some statements can be incorrect cannot be supported or regarding, e.g., the color or number of pixels of a Rubin's vase or the length of two lines in the Müller-Lyer illusion. Such statements do not involve figure-ground contrast. However, when the statements involve such contrast and they cannot be supported by the evidence, such statements are “unwarranted.”^4^

## Optical Illusions

### Examples of Optical Illusions

Optical illusions are judgments on statements that belong to the correct/incorrect genus. The judged or perceived phenomena do not involve figure-ground contrast, perspective, or context, what is at hand is the ability to verify a statement or a perception purely on empirical grounds.

**Figure 4:**
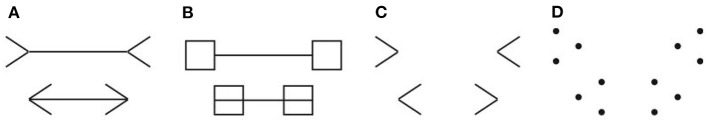
The Müller-Lyer illusion **(A)** and variations **(B–D)** (Howe and Purves, 2005, p. 1235).

**Figure 5:**
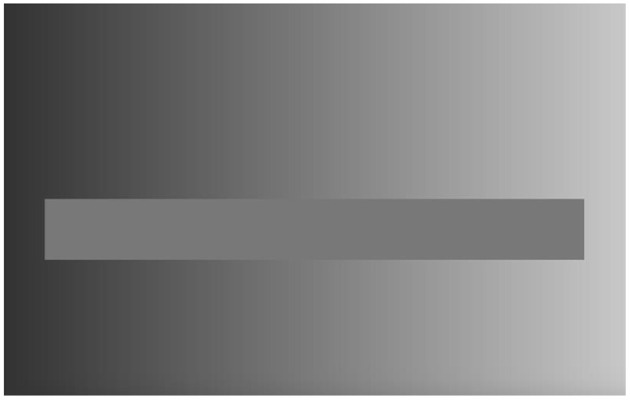
The lightness illusion (https://www.wikiwand.com/en/Optical_illusion#/google_vignette–Retrieved 2 September 2021).

The best exemplar of optical illusions is the Müller-Lyer illusion, named after the German sociologist, Franz Carl Müller-Lyer, who introduced it in 1889. Figure 4 reproduces variations of the Müller-Lyer illusion, which Howe and Purves (2005, p. 1235) introduced. In any variation, the distance between the arrow-head and arrow-tail is the same for the upper and lower lines. However, the DM judges that the upper distance is greater than the lower. Another exemplar of optical illusions is the lightness illusion (e.g., McCourt, 1982; Adelson, 2000; Bressan, 2006). As illustrated in Figure 5, the rectangular bar consists of the same shade of grayness throughout its length. However, the DM sees it as consisting of different shades, depending on the surrounding regions.

What drives the Müller-Lyer illusion is the direction of the arrow, whereas what drives the lightness illusion is the variation of the adjacent surrounding. Both drivers are benchmarks that cannot be grounds or perspectives as in Rubin's vase, what this paper aims to establish.

### How to Explain Optical Illusions?

Howe and Purves (2005) summarize the consensus on how to explain optical illusions. The DM uses a rule of perception derived from experience, but only to break down occasionally when encountering particular cases.

In relation to the Müller-Lyer illusion, the line with the arrow-tail pointing inward with the obtuse angles allows for greater space than the line with the arrow-head that is pointing outward with the acute angles. This difference must be the crucial factor, as it is the only difference between the two lines in the Müller-Lyer illusion. Deregowski (2015) shows that even when we place the two arrows in a cone, where the wide-vis-narrow cone boundary provides more or less space, such a space is irrelevant: the illusion persists. Thus, the relevant space is only the space allowed by the direction of the arrow, whether it is the arrow-tail or -head.

Hence, we may infer that the DM, from everyday experience, forms a rule of perception that is functionally similar to a rule of thumb or heuristics: a line with endings that suggest a wider space than the space entailed by a similar line usually means that the former is longer than the latter. This rule leads the DM to expect that the lines at the lower levels of Figure 4 to be shorter than the lines at the upper levels. While the rule is correct on average, it is incorrect in the Müller-Lyer illusion.

As for the lightness illusion, the issue is a bit more complicated, given that it is the outcome of many operations of vision. Bressan (2006), a vision scientist, develops the “anchoring theory” of Gilchrist et al. (1999), where she adds that valuations or impressions of the lightness of an object involve two anchors rather than one. The first anchor is how the object of assessment relates to a framework that stands alone or if the region of vision is complex, a local framework, one nested within a global framework. She defines a “framework” as a collection of groups of surfaces in the field of vision belonging together either by design, such as the black and white strips of a zebra, or by accident such as the fragments of clouds, treetops, and a flock of birds. The second anchor is how the object relates to the highest luminance in the global framework in contrast to the luminance of the local framework. Bressan argues that the lightness of an object, as assessed by the DM, is a weighted average of the values computed according to both anchors, taking into consideration whether the framework is simple or nested within a global framework. Such a weighted average, she shows, can explain various lightness illusions.

The double-anchoring theory of Bressan, typical of other theories in vision sciences, amounts to explaining the mechanics of the lightness illusion, not the why and the how of the organism seeking improvement of well-being and survival experiences such mechanics. As Zavagno et al. (2015) state, received neuroscience theories are generally insufficient:

Two friends are in a car; the passenger asks the driver: “Why are we going faster?” “Because I pressed on the pedal,” answers the driver. The answer is formally correct but not very informative. What does this have to do with illusions and neuroscience? The answer of *why* things appear as they do cannot be confined to the definition of the neural correlates of visual phenomena: the *where* issue is not a sufficient answer. In the past two decades, a lot has been written about *where* things happen in the brain, which is an important starting point that, however, does not fully address the *how* and the *why* issues. How processes take place will become clearer when neuroscientists will be able to connect single-cell responses to networks of cells and understand the communication and integration of information across networks. It is not only a question of mapping the brain and describing its architecture, but it is also a matter of understanding its functional architecture and interconnections. Neuroscience is already stepping on the path that leads to a fruitful understanding of *how* the brain processes visual information. We believe that illusions may become relevant tools in such studies, if research is not limited to finding *where* an illusion occurs in the brain but *how* and *why* it comes to be (Zavagno et al., 2015, p. 5).

The lightness illusion of the rectangular bar (Figure 5) is obviously the outcome of the variation of the shadiness of the surrounding region—and specifying the mechanics of this fact is similar to explaining why a car accelerates when the driver presses on the gas pedal. We want to know, in addition, why do organisms, whose main motive is the improvement of well-being and survival, experience damaging optical illusions. That is, why has the driver pressed on the pedal in the first place, if that increases the chances of an automobile accident? Why do organisms allow the surrounding region to act as an anchor while the surrounding region is, strictly speaking, irrelevant to judging the object of interest?

The DM apparently follows a rule of perception that necessarily entails the employment of the surrounding region, that is, its use as a benchmark, to make judgments of brightness of the bar. And the illusion, which is impervious to adjustment as long as the DM uses the benchmark, is simply the failure of the rule of deception on one occasion, when the benchmark is not acting as it should, as a uniform standard.

Similar to the Müller-Lyer illusion, even when the rule of perception fails on one occasion, the rule of perception is efficient on average. The rule of perception is the use of the adjacent region as a benchmark, allowing the DM to judge the shadiness of the bar quickly and efficiently. It is not efficient in the Bayesian updating sense. It would be efficient in the Bayesian sense if the DM totally ignored the benchmark (adjacent region) and examined the object absolutely, that is, as a case on its own without the assistance of the benchmark. However, such case-by-case examination would invite a greater cognitive cost.

The DM seems to develop the benchmark through experience. When the DM employs the implicit rule of perception, he or she can be somewhat certain of his or her judgment, namely, that the bar consists of varying shades of gray. However, in the case of a varying standard as in the lightning illusion (Figure 5), which betrays the rule of perception, the judgment is clearly incorrect. The lightning illusion arises because, on a few occasions, the benchmark should be suspended or ignored upon reflection.

However, the regret of using the benchmark is an *ex post* feeling that may not be justified *ex ante*. The suspension would not be justified *ex ante* if the cost of case-by-case measurement was higher than the expected cost of an occasional optical illusion. It would be useful to suspend the benchmark and to, instead, examine the details of the case if one was, e.g., building a bridge where mistakes are highly costly.

The proposed explanation is the bounded rationality explanation of the economist. It is implied when psychologists (e.g., Kahneman, 2011) note repeatedly the analytical similarity between cognitive illusions and optical illusions. More importantly, there is a growing and prospering field, known as “neuroeconomics,” whose promoters include neuroscientists. Neuroeconomists use cost-benefit calculations to explain neural processes and, consequently, decisions (e.g., Glimcher, 2010)^5^.

### The Critical Role of Experience

The experience of the DM is critical to the formation of the rules of perception that rely on benchmarks. Lotto generalizes the role of experience in non-human organisms such as bumblebees in experiments with collaborators (Nundy et al., 2000; Haynes et al., 2004; Lotto and Chittka, 2005; Lotto and Wicklein, 2005; Lotto, 2017). They show that bumblebees develop rules of vision because of experiencing the difference of hues of color in light of relativeness.

To clarify the terminology, Lotto and others use the term “context” to denote benchmarks such as adjacent hues of color. As suggested at the outset, “relativeness” would be a better term in order to avoid conflating rules of perception, which use “relativeness,” with Rubin's vase, which is the product of “context” strictly defined. Aside from terminology, Lotto (2017) explains that relativeness is crucial to how organisms make judgments. They do not make judgments based on some absolute or objective reproduction of their environments.

The reason is simple: The main drive of organisms, including humans, is not to discover the world as it is or to uncover the facts, which is the impetus of scientific inquiry and of the criminal justice system (Khalil, 2013b). The main drive of organisms is to see the world according to their needs: to extract nutrients, exploit resources, escape predators, and attract sexual partners. Thus, the rules of perception of space are subtle tools acquired by experience to facilitate action, procure resources, and ensure survival.

Even our assessment of space and the distance between us and a target varies, becoming smaller if other humans are present in the space. Evidently, the presence of humans changes our calculation of the chance of success in reaching the target (see Fini et al., 2014, 2020).

### Other Explanations of Optical Illusions

In light of the above, the acquired rule of perception must be effective at least on average (Howe et al., 2006). In few cases, the rule fails, generating optical illusions. As Gregory (2005) maintains, what appears as an optical illusion is nothing but the failed application of a rather efficient rule of perception.

The view of Gregory entails that optical illusions are departures from what is real. Indeed, Gregory (2009) draws an explicit dichotomy between the real and the illusory. This dichotomy, however, did not stand without a challenge. Rogers (2014), for example, questions the dichotomy of what is called “veridical perception,” that is, non-illusory perception of objective reality, and “illusory perception.” For him, it is impossible to draw a line between them, as no one knows what is really out there. Whatever we see, including what is non-illusory perception, is the construct of the senses. For Rogers, all perception is the outcome of how our biological system processes light, smell, touch, taste, and sound.

This argument was made long ago by British empiricist philosophers such as Berkeley (2009). This paper is not the place to tackle the argument of Rogers in its detail—not to mention the arguments of the British empiricists. It is sufficient, however, to recall the argument of Lotto and his collaborators (Nundy et al., 2000; Lotto and Chittka, 2005; Lotto and Wicklein, 2005; Lotto, 2017), namely, that the primary purpose of organisms is not to know “what is the real world.” They rather form rules of perception where such rules, based on experience, are tools that extract nutrients, fend off predators, and so on.

While the rejection of Rogers of the dichotomy of Gregory is convincing, it should not entail the rejection of the difference between optical illusions and non-illusory perception. Once we view non-illusory perception as based on rules acting as tools for survival, rather than as motivated by the pursuit of knowledge for its own sake to replicate “what is out there,” we can view perceptual illusions as the occasional failure of such tools. In this manner, there is no dichotomy between veridical perception and illusory perception. There is only the chronicle of a tool, that is, a rule of perception: how it originates, how it succeeds, and how it occasionally fails.

There are other explanations of the rules of perception and, correspondingly, of perceptual illusions (see Purves and Lotto, 2010). Zavagno et al. (2015) provide a useful guide by offering a succinct taxonomy. They identify three classic approaches of vision sciences—which entail three different explanations of optical illusions:

1. **Ecological approach:** It simply regards vision, or any perceptual sensation, as a replica of the environment. Thus, DMs perceive things as they are. And when confronted with perceptual illusions, this approach explains them away as the outcome of poor information.
2. **Gestalt approach:** It regards perception as inevitably egocentric in the sense that a person organizes the sensory data in a way that meets his or her own needs. For the gestalt approach, the notion of veridical perception is irrelevant in the first place. There is no special urgency to explain perceptual illusions since all perception involves a gestalt, i.e., it cannot ever represent the world.
3. **Cognitive approach:** It regards perception as biased as a result of prior experience, prejudice, and logical reasoning. So, two DMs with different prejudices may process the same data differently. As for perceptual illusions, the cognitive approach regards them as errors that can be explained as the outcomes of the entwined cues. Researchers can use the errors to study how DMs employ logical reasoning and how such reasoning might fail in the face of confusing cues.

All three approaches basically fail, each in its own way, to radically distinguish the two reference points. According to the core hypothesis of this paper, contexts that generate Rubin's vase do not lie along the same continuum that accommodates rules of perception that might occasionally give rise to optical illusions.

### Contexts vs. Rules of Perception

We can now identify many reasons why rules of perception cannot function as contexts:

1. The DM can simultaneously see the two ends of the bar in the lightning illusion (Figure 5), where one end is lighter than the other, while the DM cannot simultaneously see the two alternative figures in Rubin's vase, the alternative locations of the red ball in the Necker cube, etc.
2. If one increases the darkness of the benchmark at one end relative to the other end (Figure 5), the difference of shadiness of the bar would become accentuated. In comparison, if we increased the dark pigments in Rubin's vase, all else being equal, there would be no impact on whether one gestalt appeared more frequently than the other.
3. If we replaced the benchmark in Figure 5 with a picture of the New York skyline, the optical illusion would vanish. In comparison, the alternative gestalt in Figure 1 does not depend on the nature of the pigmented pixels. If we replaced the pigmented pixels with the New York skyline, the alternative figures would persist. If we replaced the non-pigmented pixels with the New York skyline, the alternative figures would persist. This supports the thesis that the ground (context) in Rubin's vase cannot be seen as a rule of perception but rather, must be seen as a perspective against which the DM can make sense of the figure.
4. As mentioned above, once the DM realizes that the bar is of homogenous shadiness, he or she continues to see the bar as of heterogeneous shadiness. Likewise, while the DM realizes that the lines in the Müller-Lyer illusion are of the same length, he or she continues to see them as of unequal lengths. Nonetheless, the DM must agree that both perceptions are incorrect. In contrast, the DM does not find the alternative figures, particularly in the plain Rubin's vase, to be incorrect.

## Framing Effects

Similar to Rubin's vase, the DM does not find the alternative choices in response to the loss/gain frame experiments to be incorrect. One famous illustration of the loss/gain frame effect is the Asian disease experiment of Tversky and Kahneman (1981) already mentioned above.

To recall the discussion above, the loss/gain frame effect involves a more or less fixed ground. This is similar to the detailed Rubin's vase—where the figure is either gold-shaded or detailed with facial hair. Thus, the DM would most likely judge the effect to be either a vase or two opposed faces in a profile, depending on the details. Similarly, with the loss/gain frame, the DM would be more likely to choose one option over the alternative. Thus, as mentioned above, we need a between-subject experimental design to verify whether subjects who choose one choice in the framing effect would be ready to correct their choices once told about the alternative context and, consequently, the alternative choice.

Leboeuf and Shafir (2003) test exactly such a design using various examples replicating the canonical Asian disease setup. In example after example, DMs do not correct their choices once told, after the experiment, that another group has made a different choice, given the alternative context. DMs, basically, do not consider their choices to be an error—contrary to DMs subjected to cognitive illusions as shown below.

To revisit the story of the marathon runner who won a “second rank,” the framing effect would be negative if the winner used “first rank” as the context. If the runner employed, instead, the “third rank” as the context, the framing effect would be positive. If the runner chose the “third rank” as the context, he or she would not normally consider the alternative context and the consequent framing effect to be incorrect.

Economists noticed long ago, at least as far back as Duesenberry (1949, see Khalil et al., 2021), the importance of employing the average income of a peer group as the context of evaluating the income of an individual—what they call the “relative income hypothesis.” Namely, the framing effect is positive upon contrasting the income of an individual against a lower income within a peer group and negative against a higher income within a peer group. Indeed, the DM may manipulate the context, imagine diverse counterfactuals, etc., to increase happiness. However, the full implication of such manipulation, once we also take into consideration how aspiration is important to happiness, is rather involved and falls outside the scope of this study (see Khalil, unpublished).

## Cognitive Illusions

### What Are Cognitive Illusions?

Cognitive illusions are ubiquitous. Tversky (1972b); Kahneman and Tversky (1973); Tversky and Kahneman (1974) started their research program by focusing on one example of cognitive illusions, namely, what happens when the availability heuristics fails. They quickly generalized their research program to include other phenomena such as the representativeness heuristics, the Linda Problem, and the base-rate neglect. These phenomena and others, such as the Wason Selection Task (Wason and Evans, 1974), belong to the same genus, namely, that people consciously or subconsciously follow rules of thumb, generalizations, first impressions, anchors, first-evoked memory, or in short, “heuristics” —which are certain to occasionally fail, thereby generating cognitive illusions.

To explain the origin and functions of heuristics, Kahneman resorted to dual process theory in his later work (Kahneman, 2011; Khalil and Amin, unpublished). To be clear, there are many versions of dual process theory (see Evans, 2009). The version of Kahneman (2011) differentiates between the heuristic/intuitive system of reasoning from the analytic/deliberative process of reasoning. Following the convention (e.g., Sloman, 1996; Stanovich and West, 2000), Kahneman uses the term “System 1” to denote the heuristic/intuitive process and “System 2” to denote the analytic/deliberative process. While the heuristic System 1 relies on ready-made stereotypes and heuristics, the analytic System 2 engages with the facts of the specific case and is hence vigilant.

Why do choices rely on two systems? The vigilance of System 2 involves a cognitive cost. In many cases, it is more efficient to follow a rubric or heuristics than undertaking a costly cognitive deliberation. This is the case even when the heuristics could invite a costly mistake. Such a mistake appears as a behavioral bias or, generally, a cognitive illusion.

Thus, a cognitive illusion is tolerated as long as, in light of *ex ante* calculations, the occasional expected cost of a mistake is lower than the cost of undertaking vigilant case-by-case deliberations. That is, even when heuristics fails *ex post*, it behooves the DM to continue employing the heuristics if *ex ante* calculations deem that the expected benefit of successes, on average, exceeds the benefit of case-by-case examinations.

Thus, heuristics is an efficient tool or what we may call “technology.” Economists call such a tool “second best” in the sense that the “first best” is the judgment based on a detailed examination of the facts. If heuristics is efficient, the “first-best” solution is not optimal.

This notion of the “second best” is in line with the concept of the standard economist of bounded rationality and with the concept of Kahneman of mental economy. It differs from the ecological explanation of heuristics, also known as the “fast-and-frugal” research program (e.g., Hertwig et al., 2013), which was spearheaded by Simon (1957), and advanced by Gigerenzer and Selten (2002; see also Gigerenzer et al., 2011). This study cannot discuss it. It is sufficient, however, to note that it considers heuristics as “second best” in the sense that DMs cannot undertake maximization. Maximization is merely a fiction of the mathematicians. DMs rather stumble on a habit, a norm, or heuristics, and they adopt it as long as it is good enough, what Simon (1957) calls “satisficing.” For him, the DM does not choose the optimum heuristics because it does not exist. The DM adopts heuristics as long as it “satisfices” a given level of needs.

Clarifying further, the notion of “second best” that is used here, that is, along the later work of Kahneman (2011) and the “bounded rationality of the economist,” differs from the early research program of Tversky and Kahneman, which they called “heuristics and biases.” In their early work, they focused on uncovering behavioral biases that deviate from the predictions of standard rational choice theory. However, as the later work of Kahneman (2011) implies, their early work seems to have erected a straw man version of rational choice. Once rational choice theory is enriched with cognitive cost, these biases are, rather, the necessary costs of the occasional failures of otherwise efficient heuristics.

These occasional failures, i.e., cognitive illusions, rather affirm the efficiency of heuristics under focus. These cognitive illusions parallel optical illusions, where a rule of perception that is, on average, efficient may fail on one or a few occasions in deciphering the object of assessment. This implies that DMs do, and should, abandon heuristics or a rule of perception only when the cost of the illusions starts to surpass the cost of case-by-case examination.

### Example: Base-Rate Neglect

The tendency of the DMs to regularly ignore the base rate in their decisions illustrates why heuristics is generally efficient in the second-best sense (for a discussion of other kinds of heuristics, see Appendix 1, from the author or available as “Supplementary Material” at the end of the paper). One famous example of base-rate neglect is the set of lab experiments on taxi companies and their possible involvement in a hit-and-run accident (see Koehler, 1996; Mandel, 2014). DMs were asked about the likelihood that the culprit driver is from the blue taxi company, as opposed to the green taxi company, the only two companies operating in the city. DMs were given two facts. First, blue taxis make up only 15% of all taxis in the city. Second, there is an eyewitness who saw a blue taxi involved in the hit-and-run. But when examined to see the reliability of the witness under similar circumstances of the accident, the court finds that he is 80% reliable.

Surprisingly, most DMs have guessed that the likelihood is 80% that a blue taxi was the culprit. DMs have neglected the base rule, namely, the low frequency of blue cabs in the city. If DMs follow Bayes' rule, where they should take into consideration the low frequency of blue cabs, the likelihood that a blue cab was involved in an accident is 41%.

However, as Ajzen (1977) explains, DMs do take into consideration the base rate if the frequency of the taxis in the city is linked *causally* to the frequency of committing traffic accidents. When DMs undergo another treatment, in which they were informed that green cabs were involved in 85% of the accident while the blue cabs in 15%, i.e., when they were given more than just list the statistical fact of their relative size, DMs reduced the likelihood that a blue cab involved significantly, from about 80 to 60%.

The finding of Ajzen (1977) still means that DMs did not fully follow Bayes' rule. However, lowering the likelihood that a blue cab is involved in the accident is significant and, indeed, good news to Kahneman (2011). As Kahneman explains (2011, Ch. 6), the human mind is “lazy”; it chooses the easiest method or habit to decide. When the same information about the sizes of the taxi companies is placed in a *causal* mechanism behind accidents, the human mind finds it easier to reason using sheer statistical facts, that is, easier to attend to the base rate.

In addition, people in the lab were asked about the likelihood of a culprit with no real responsibility, they are not, for example, making judgments as a jury. The judgment as a lab subject has no serious consequences. Thus, to economize on cognitive efforts, DMs are not ready to expend cognitive cost and challenge the implicit assumption that two taxi companies are equal in size.

## Payoffs

The disentanglement of Rubin's vase from optical illusions harbors many payoffs. To mention three briefly:

1. **Happiness studies:** The proposed distinction helps us deepen the theory of human satisfaction. One may distinguish between “happiness,” on one hand, and what economists call “well-being” or “utility,” on the other. It seems that “happiness” is a kind of satisfaction that depends on the context as it amounts to the evaluation of well-being vis-a-via a context. Meanwhile, well-being is a kind of satisfaction that depends solely on pecuniary cost-benefit utility, which is context free. Such pecuniary utility is the sole basis of heuristics as this paper shows. It is important to distinguish happiness from well-being to solve the well-being-happiness paradox (Khalil, 2019; Khalil, unpublished). In contrast, the main workhorse concept in happiness studies—namely, “subjective well-being” —has been expressly coined by Diener (1984, 2009) to obliterate the difference between happiness and well-being. The conflation of happiness and well-being is an obstacle in the way to solving the well-being-happiness paradox.
2. **Moral judgments:** The proposed distinction shall shed light on the root difference between two kinds of moral judgments. There are moral judgments that use property rights and promises as “facts” against which one can judge the choices of DMs. These judgments are geared to promote social welfare (well-being), similar to the function of heuristics (rules of thumb) in personal choice. In contrast, there are moral judgments using visions and ideologies as criteria against which one can judge the choices of DMs. These judgments are geared to advance a vision of the good life and good society, where such visions act as a context of the assessment of actual well-being.
3. **New philosophy of science:** The proposed distinction has an implication with respect to a major controversy in the philosophy of science: the debate between the logical positivists, such as the followers of Karl Popper, and their critics, such as the followers of Thomas Kuhn. The positivists stress that scientists and humans, in general, ground their beliefs on facts—similarly to how they ground heuristics on facts. In contrast, the critics stress how non-verifiable contexts, i.e., “paradigms,” underpin scientific revolutions and the development of the everyday scientific activity. If we are successful in showing heuristics, which is based on facts, can coexist with contexts, which are not grounded on facts, we can see that the positivists and their critics are disagreeing because, indeed, each camp focuses on a different space of cognitive activity (see Appendix 2, from the author or available as “Supplementary Material” at the end of the paper).

## Conclusion

This study shows why Rubin's vase cannot be an optical illusion. Rubin's vase involves figure-ground contrast, calling for the role of perspective (context) in making meaning of sense data. In the case of Rubin's vase, DMs see alternative figures that can neither be correct or incorrect. At most, in light of extra data, one figure could become more warranted than the alternative. The extra data can never, by definition, invalidate the alternative perspective, i.e., the alternative figure.

In contrast, optical illusions arise when DMs use rules of perception incorrectly on some occasions. Despite the incorrect use, these rules are efficient on average. DMs are generally ready to correct the mistaken use. That is, there are correct and incorrect applications of the rules of perception—unlike the alternative figures in Rubin's vase.

The disentanglement of Rubin's vase from optical illusions offers a great opportunity. It allows us to see why the framing effects differ radically from cognitive illusions. As Table 1 at the outset lays the juxtaposition, Rubin's vase and framing effects are the outcomes of the use of perspectives or contexts to make sense or to reflect upon pertinent facts. In contrast, optical illusions and cognitive illusions, which are the failures of rules of perception or rules of thumb, are the outcome of the use of relativeness to facilitate efficient judgments. While rules of perception and rules of thumb (heuristics) economize on scarce cognitive resources, DMs may occasionally apply them incorrectly. DMs, by definition, move quickly to recognize these failures as illusions, ready to expose them as incorrect judgments.

The difference between rules of perception and rules of thumb (heuristics), on one hand, and gestalts and framing effects, on the other, lies deep. When the reference point is the loss/gain frame, analogously to the ground in Rubin's vase, the framing effect expresses how the DM feels about his or her well-being, accomplishments, or realized wealth. This invokes issues related to non-pecuniary satisfaction (happiness) that deserve a special study (Khalil, unpublished). When the reference point is heuristics on how to categorize an object or an event, the DM economizes on cognitive resources. This invokes issues related to standard economics, that is, to the study of pecuniary satisfaction, the standard concept of utility.

## Author Contributions

The author confirms being the sole contributor of this work and has approved it for publication.

## Funding

Open Access funding was provided by the Qatar National Library.

## Conflict of Interest

The author declares that the research was conducted in the absence of any commercial or financial relationships that could be construed as a potential conflict of interest.

## Publisher's Note

All claims expressed in this article are solely those of the authors and do not necessarily represent those of their affiliated organizations, or those of the publisher, the editors and the reviewers. Any product that may be evaluated in this article, or claim that may be made by its manufacturer, is not guaranteed or endorsed by the publisher.

## Footnotes

^1^Incidentally, M. C. Escher (1898–1972) adopts Schröder staircase in his “Relativity” and “Convex and Concave” lithographs (see Bool et al., 1982).

^2^As a historical note of irony, the term “bounded rationality,” which is now part of the toolbox of neoclassical economics, was coined originally by Simon (1957). He coined it to express the idea that DMs cannot maximize; they are rather bounded in their rationality because they are involved in “satisficing,” as explained below. Given that the term was hijacked by neoclassical economists, i.e., to denote optimization, Simon (1976) proposed another term “procedural rationality” as contrary to the bounded rationality of the economist.

^3^Furthermore, Kahneman is not careful with his characterization of heuristics. For instance, Kahneman (2011, pp. 372–373) characterizes mistaken heuristics—namely, in judging which of the two contrasts of automobiles affords greater saving of gasoline—as a “frame effect”.

^4^Critics of positivist philosophy of science, such as Dewey and Bentley (1949), tend to think that “all” statements are unwarranted, as if they all necessarily involve perspective, i.e., figure-ground contrast (see Khalil, 2003, 2010). However, the bulk of the philosophical literature on vision and perception goes in the opposite direction, namely, treating “all” statements as the subject of correctness verification, i.e., empirical verification. This leads the literature to slip and regard Rubin's vase as the product of information ambiguity and consequently, to lay Rubin's vase and optical illusions along a continuum (see Macpherson, 2006, 2012; Silins, 2019). Such a tendency in the philosophical literature on vision is driven by a more fundamental and old concern of philosophers, the validity of beliefs in whether the world exists (e.g., Searle, 1992; Smith, 2002, 2008).

^5^One approach worth mentioning is the application of cost-benefit calculation to explain bodily movement, where the vigor of the DM is based on the expected benefit and the cost of physical effort (Shadmehr and Ahmed, 2020).
