# Supplementary material for: Why Does Rubin's Vase Differ Radically From Optical Illusions? Framing Effects Contra Cognitive Illusions
Source: Front Psychol. 2021 Sep 21;12:597758. doi: 10.3389/fpsyg.2021.597758 (PMC8490627; doi:10.3389/fpsyg.2021.597758)
Supplement: Supplementary file 1 [file Table_1.DOCX]

**Why does Rubin’s Vase Differ Radically from Optical Illusions?**

**Framing Effects *contra* Cognitive Illusions**

**Elias L. Khalil**

**Appendix 1:**

**Examples of Cognitive Illusion**

Cognitive illusions appear with the occasional failures of other heuristics such as the availability heuristic, representativeness heuristic, the Linda Problem, over-inference (the fallacy of the law of small numbers), Kahneman’s distinction between “remembering utility” and “experience utility,” and the Wason Selection Task (see Khalil & Amin, 2021).

With respect to the availability heuristic, one good example is the tendency of DMs to judge that words starting with the letter “r” at the first position is greater than words with “r” at the third position. In fact, there are more words with “r” at the third position than words with “r” at the first position. However, given the cognitive cost, memory tends to remember more words that start with a specific letter than with words that have this letter at a second or third position. Such a heuristic can be explained via Kahneman’s mental economy, that is, that it is efficient. However, it involves mistakes, that is, cognitive illusions as in the case of the letter “r.”

With regard to the representativeness heuristic (see Kahneman & Tversky, 1972, 1973), a good illustration is the case of “Steve” the librarian, which Kahneman (2011, pp. 6-7) recounts at the outset of his book. DMs seem to group objects on the basis of simple resemblance to a prototype that already exists in their mind. DMs tend to think without deliberation that the prototype is the best illustration of a particular object. When subjects were told that Steve is quiet, attentive to details, and bookish, most of them decided that he is more likely to be a librarian than a farmer. The subjects neglected the base rate, namely, there are 20 times more farmers than librarians.

The use of a prototype or a representative is an economical way of judging the profession of individuals, classifying an object to a specific class, and so on. However, on some occasions, as the judgment of Steve’s most likely profession, DMs make a mistake, incurring a cognitive illusion.

As for the Linda Problem (Tversky & Kahneman, 1983), it combines the representative heuristic with error in logical reasoning. On one hand, DMs seem to have used the representative heuristic when they regarded Linda to be most likely a “feminist,” as feminism seems to be the prototype that expressed Linda’s social commitments when she was a college student. On the other hand, when asked about Linda’s most likely profession, most DMs chose the likelihood that Linda is a “bank-teller/feminist” to be higher than the likelihood “bank teller.” This is a violation of logical reasoning, as the category “bank teller” is broader than “bank-teller/feminist.”

There are many interpretations of the Linda Problem, which this paper cannot review (see Kahneman, 2011). It seems that DMs focused on the salient issue, namely, what is Linda’s prototype, that is, feminism, given the lengthy description of her commitments. The issue of her profession arose only in response to the arbitrary choices of the question posed. That is, the issue of her profession is a secondary focus—given of course that “feminist” is not a profession. Therefore, the “feminist” category seemed to be an independent category that can be (wrongly) added it to “bank teller” category, to conclude incorrectly that both categories are larger than the “bank teller” category. It seems that the representative heuristic, “feminism,” guided the DMs’ choice leading to the cognitive illusion when the secondary category regarding the profession was added.

As for the over-inference (the fallacy of the law of small numbers), DMs tend to generalize beyond what the data allows them. A DM tends to build a stereotype, for example, of “Thai people” based on three or four encounters with people with Thai ethnicity. Such a generalization, nonetheless, is efficient, given that DMs are not conducting scientific research in which they have to optimize the use of data for the sole purpose of correct inference. As argued elsewhere (Khalil, 2013), DMs are engaged in practical pursuits, whereas an overgeneralization is more beneficial on average than the cost of some mistaken judgment, that is, cognitive illusion. Of course, when the stereotype is critical for major investment decisions or hiring decisions, the cost of the mistake might exceed the expected benefit from the stereotype. The mistake is no longer a cognitive illusion, but rather a call to adjust the heuristic itself (See Kahneman, 2011).

As for Kahneman’s distinction between “remembering utility” and “experience utility,” it is very useful as it clarifies what DMs use as facts when they make forward-looking decisions. It is an efficient heuristic for DMs to use remembering utility, rather than experience utility, as the DM needs to expend high cognitive effort in adding the pleasures or pains of each moment of the experience. It is less costly, that is, for the DM to calculate the benefit of a past experience based on the last episode, which is easy to remember, and the peak episode, which stays in memory without effort given its relative sharpness. However, on some occasions, the heuristic fails, that is, the DM falls into cognitive illusion, when experience utility greatly departs from remembering utility. However, the heuristic remains efficient if, *ex ante,* the saved cognitive effort of computing experience utility exceeds the occasional cost of cognitive illusions.

For the final example, the Wason Selection Task (Wason & Evans, 1974), DMs are asked a question of logic: what cards out of four cards do we need to turn over to test a hypothesis that a particular color, say “red,” on one side means necessarily a particular number, say “4.” Most DMs chose the “red” and the “4” cards as the necessary ones to test the hypothesis, when in fact it is the “red” and the “3” cards. MDM’s choice is rather predictable. It is the most efficient answer given that the question has primed the DMs to pick these two cards—and given that the reward may not justify extensive mental effort of the logical reasoning at hand. Here, a simple heuristic, namely, pick the cards that the experimenter has spelled out, turns out to be a mistake, a cognitive illusion. Nonetheless, the heuristic may, *ex ante,* continue to be efficient from the standpoint of Kahneman’s mental accounting.

In all these phenomena of heuristics, the bounded rationality explanation or Kahneman’s mental economy entails that once the DM is alerted about the need to deliberate on the correct judgment, that is, to avoid the use of the heuristic, the DM would attend to the details of the task at hand. Indeed, even when the heurist continues, *ex ante,* to be efficient despite the just experienced failure (cognitive illusion), the DM is more ready to correct the mistake, as obviously the cases of Steve the librarian, the Linda Problem, the Wason Selection Task, and so on.

**Appendix 2:**

**Rubin’s Vase, Optical Illusions and the Philosophy of Science**

The difference between “un-warranted” and “incorrect” statements has great implication for the philosophy of science. Indeed, Dewey and Bentley (1949; see Khalil, 2003, 2010, 2011, 2013) stressed the difference between the two statements. One may register that the statement “the invasion of the U.S. of Iraq in March 2003 did not violate international law” to be incorrect. It is simply contrary to a norm-based fact, a fact that is created according to an established norm. Or some legal scholars could argue that the statement is correct. Irrespective of the final finding, it is a judgment of correctness vs. incorrectness.

However, “un-warrantness” is related to opinion, perspective, or framing. For example, one cannot argue that the statement “the U.S. invasion of Iraq in 2003 served the national interest of the US” to be correct or incorrect. Even if sufficient facts took place, one can narrate them within a perspective that affirms, of disconfirms, the statement. . One could at best argue for the warrantness or un-warrantness of the perspective.

Put differently, the criterion “what is the national interest” is a matter of perspective. Whatever new facts that emerge, whether to show that greater benefits or costs of the invasion, such facts only buttress a perspective without invalidating the alternative as incorrect.

The difference between incorrectness and un-warrantness is at the core of the debate in the philosophy of science between the positivist tradition and the new philosophy of science. For the positivist approach, as matured in Karl Popper’s falsificationist theory, *all* statements can be subjected to the correct/incorrect criterion. For the new philosophy of science, epitomized in the work of Thomas Kuhn, many statements are rather the subject of the warranted/un-warranted criterion. For the new philosophy of science, statements are rather based on paradigms, where paradigms roughly correspond to perspectives or grounds that allow the scientist to frame the facts. Therefore, for Kuhn and the new philosophy of science, scientific theory is ultimately a narrative, that is, it is based on the perspective that identifies a ground or a context. Hence, scientific theory, contrary to Popper, cannot be evaluated according to the correct/incorrect criterion (Khalil, 2013).

Similarly to Kuhn’s position, Rubin’s vase entails that judgments cannot be along the Popperian correct/incorrect criterion. When one sees a vase or the two-opposed-faces-in-profile, both are perspectives that cannot be correct or incorrect. Even if one claims that he sees two elephants or three valleys, it is a matter of perspective that cannot be correct or incorrect. What is at issue is not whether the shape is a vase, the two-opposed-faces-in-profile, an elephant, or three valleys. What is at issue is whether such one judgment can rule out the others. In the case of framing or Rubin’s vase, the judgment of X shape cannot rule out the other shapes insofar as the judgments arise from the change of ground. Thus, the judgments cannot be made along the Popperian correctness/incorrectness criterion. Each judgment in Rubin’s vase is the outcome of ground or perspective that is not even an empirical issue. At best, one can assert that the two-opposed-faces-in-profile, say, is un-warranted, as in Figure 2, the gold-shaded Rubin’s vase.

Dewey and Bentley, for their part, regarded all statements as bounded by perspectives or context and, hence, totally rejected the possibility that some statements can be subjected to the correct/incorrect criterion. They advocated exclusively for the the warranted/un-warranted criterion. As just argued, however, not all statements or judgments involve perspective or figure-ground contrast. For example, when asked about adding 9 plus 23, the DM applies the correctness/incorrectness criterion. The DM might answer incorrectly “31.” Likewise, when asked about the temperature of a cup of tea, the DM may answer incorrectly “very hot.” The incorrect answer might have risen because the DM failed to take account that he had an abnormal benchmark: he or she just drank iced water. Once the DM is alerted about the abnormal benchmark, he or she would usually correct the mistaken judgement.

Stated differently, it is the case that an immediate experience, for example, of the brightness of light, influences the DM’s judgment of the luminousness of the gold color. Likewise, a preceding experience, for example, of sweetness, influences the DM’s judgment of the taste of a peach or an apple. Moreover, a habitual consumption of spicy food influences the DM’s judgment of the spiciness of food. But prior experience, habit, and so on, that act as benchmarks that help or hinder the DM in judging relative luminosity of light, sweetness, or spiciness of objects should not be conflated with the “ground” that define Rubin’s vase. Benchmarks allow the DM to reach correct, incorrect, or partially correct judgments. As shown next, a benchmark, hence, differs from the ground in Rubin’s vase, where the ground permits the DM to reach warranted or un-warranted judgments.

**References**

Dewey, John and Arthur Bentley. *Knowing and the Known*. Boston: Beacon Press, 1949.

Kahneman, Daniel. Thinking, Fast and Slow. New York: Farrar, Straus and Giroux, 2011.

________ . "The Psychology of Preferences." Scientif­ic American, 1982, 246, pp. 160-73._and Amos Tversky. “Subjective Probability: A Judgment of Representativeness.” *Cognitive Psychology*, 1972, 3:3, 430–454.

________. “On the Psychology of Predictions.” *Psychological Review*, July 1973, 80:4, 237-251.

Tversky, Amos and Daniel Kahneman. “Extensional versus Intuitive Reasoning: The Conjunction Fallacy in Probability Judgment.” *Psychological Review*, 1983, 90, 293–315.

Khalil, Elias L. “The Context Problematic, Behavioral Economics and the Transactional View: An Introduction to `John Dewey and Economic Theory’.” *Journal of Economic Methodology*, June 2003, 10:2, pp. 107-130.

________. “The Bayesian Fallacy: Distinguishing Internal Motivations and Religious Beliefs from Other Beliefs.” *Journal of Economic Behavior and Organization*, August 2010, 75:2, pp. 268-280.

________. “Rational, Normative and Procedural Theories of Beliefs: Can They Explain Internal Motivations?” *Journal of Economic Issues*, September 2011, 45:3, pp. 641-664.

________. “Practical Beliefs vs. Scientific Beliefs: Two Kinds of Maximization.” *Theory and Decision*, 2013, 74:1, pp. 107-126.

________ and Azzam Amin. “Cognitive Illusions, Physiological Malfunctions: The Dual Process Theory and Cognitive Economy.” A paper submitted for publication, 2021.

Wason, P.C. and M.S.F. Evans. "Dual Processes in Reasoning?" *Cognition*, 1974, 3:2, 141–154.
